# Probiotics as the live microscopic fighters against *Helicobacter pylori* gastric infections

**DOI:** 10.1186/s12876-021-01977-1

**Published:** 2021-10-20

**Authors:** Masoud Keikha, Mohsen Karbalaei

**Affiliations:** 1 Department of Microbiology and Virology, Faculty of Medicine, Mashhad University of Medical Sciences, Mashhad, Iran; 2 Student Research Committee, Mashhad University of Medical Sciences, Mashhad, Iran; 3 Department of Microbiology and Virology, School of Medicine, Jiroft University of Medical Sciences, Jiroft, Iran

**Keywords:** Gastric cancer, *Helicobacter pylori*, *Lactobacillus*, Peptic ulcer, Probiotic

## Abstract

**Background:**

*Helicobacter pylori* (*H. pylori*) is the causative agent of stomach diseases such as duodenal ulcer and gastric cancer, in this regard incomplete eradication of this bacterium has become to a serious concern. Probiotics are a group of the beneficial bacteria which increase the cure rate of *H. pylori* infections through various mechanisms such as competitive inhibition, co-aggregation ability, enhancing mucus production, production of bacteriocins, and modulating immune response.

**Result:**

In this study, according to the received articles, the anti-*H. pylori* activities of probiotics were reviewed. Based on studies, administration of standard antibiotic therapy combined with probiotics plays an important role in the effective treatment of *H. pylori* infection. According to the literature, *Lactobacillus casei*, *Lactobacillus reuteri*, *Lactobacillus rhamnosus* GG, and *Saccharomyces boulardii* can effectively eradicate *H. pylori* infection. Our results showed that in addition to decrease gastrointestinal symptoms, probiotics can reduce the side effects of antibiotics (especially diarrhea) by altering the intestinal microbiome.

**Conclusion:**

Nevertheless, antagonist activities of probiotics are *H. pylori* strain-specific. In general, these bacteria can be used for therapeutic purposes such as adjuvant therapy, drug-delivery system, as well as enhancing immune system against *H. pylori* infection.

## Background

*Helicobacter pylori* (*H. pylori*) is a gram-negative, motile, helical and microaerophilic microorganism that is considered as one of the most successful pathogens due to persistent infection in human stomach [1]. The global prevalence of this bacterium is high, so that according to the latest statistics *H. pylori* has colonized the stomachs of 4.4 billion people worldwide [2]. There is ample evidence that *H. pylori* is the etiologic agent of both gastric (gastric malignancy, peptic ulcer, chronic gastritis) and extragastric diseases [3–5]. Depending on the geographical area, the rate of infection with this pathogen varies; frequency of infection with this bacterium is associated with several factors such as virulence factors (e.g. CagA and VacA) and socioeconomic status, for example the rate of infection in some parts of Africa is close to 100% [6]. According to the literature, post-treatment re-infection is common in low-income countries with poor public health policy [7]. Basically all patients infected with this bacterium should be treated; complete eradication of *H. pylori* improves peptic ulcer and mucosa-associated lymphoid tissue (MALT) lymphoma, as well as reduces the risk of gastric cancer and autoimmune liver disease [8–10]. The most common problems facing gastroenterologists include, (1) antibiotic-resistance phenomenon, (2) persistence of bacteria in latent status, (3) degradation of antibiotics in acidic gastric conditions, (4) re-infection especially in regions with high prevalence, (5) adverse side effects of antibiotics such as diarrhea, nausea, vomit, and abdominal pain, (6) rapid metabolization of antibiotics due to CYP2C19 enzyme, (7) poor compliance of multiple antibiotics [11–13]. In recent years, antibiotic resistance (with high divergence) has led to increased therapeutic failure in eradicating *H. pylori* with current regimens [14, 15]. In the early 1990s, the eradication rate of the standard triple therapy was more than 90%, however, in recent decades, the effectiveness of this regimen has dropped to less than 70% [16–18]. According to the World Health Organization (WHO) report, the rate of resistance to clarithromycin and metronidazole ranged 14–34% and 20–38%, respectively [19]. Graham et al. suggested that the therapeutic regimens with less than 80% efficacy are considered as treatment failure [20]. Recently, adjuvant therapy with probiotics has received much attention as a new strategy to increase the success of anti-*H. pylori* therapy [15]. Probiotics are a group of bacteria that confer various health benefits to the host [21]. Intestinal colonization with these microorganisms maintains the integrity of the mucosal immune system and inhibits the side effects associated with antibiotic use [21, 22]. Probiotics are used for purposes such as treating diarrhea and preventing allergic reactions [23]. In vitro studies have shown that some probiotics particularly *Lactobacillus* spp. possess anti-*H. pylori* activities [24]. García et al. found that co-existence of *Lactobacillus* and *H. pylori* in patients with severe gastrointestinal diseases was significantly lower than control subjects (without clinical symptoms); colonization of *Lactobacillus* spp. in stomach leads to several events such as reducing gastritis, promoting mucin regeneration, as well as downregulating gene expression in *cag* pathogenicity island [25]. Therefore, probiotic supplementation is considered as one of the promising solutions for the treatment of *H. pylori* infection in symptomatic patients [15]. Based on studies, the use of probiotics as a supplement in addition to standard antibiotic treatment significantly improves the eradication rate of *H. pylori* infection compared to the administration of antibiotics alone [26, 27]. The main purpose of this study was to provide an overview of the benefits of using probiotics in the treatment of *H. pylori* infection.

## *H. pylori* antibiotic resistance and current treatment regimens

### First-line therapy

According to European Helicobacter and Microbiota Study Group (EHMSG) guidelines, triple therapy is still recommended as the first-line treatment for *H. pylori* infection in areas with low clarithromycin rate [28]. Increasing clarithromycin resistance leads to reduce the eradication rate of clarithromycin-containing triple therapy, for example in Argentina cure rate is estimated at 75% [29]. The situation in South Korea is even worse, so that based on the duration of treatment, the cure rate with this regimen has been estimated at 64% and 66% for 7 and 14 days, respectively [30]. According to the literature, clarithromycin resistance rates are 10.6–25%, 16%, and 1.7–23.4% in North America, Japan, and Europe, respectively [30–33]. On the other hand, metronidazole resistance is also increasing, so that the resistance in European and African countries is 17–44% and 100%, respectively [34–36]. Recently, Yao et al. showed that the rate of infection eradication in type 2 diabetic patients is up to 74% [37]. Bismuth quadruple therapy, a complex regimen containing proton pump inhibitors (PPIs), bismuth salt, tetracycline, and metronidazole is also recommended as second-line (or even first-line) in high clarithromycin resistance areas [38]. In accordance with multicenter randomized controlled trials (RCTs)‏, curing rate of bismuth quadruple therapy is significantly higher than the standard triple therapy (90.4% vs. 83.7%) at the same time (for 14 days) [39]. However, in a meta-analysis study, Luther et al. evaluated nine RCTs, and found that the eradication rate of infection in patients receiving bismuth quadruple therapy was the same as those who had received clarithromycin triple therapy (78.3% vs. 77%) [40]. But it should be noted that bismuth citrate is harmful to human health, so this drug (or even tetracycline) is contraindicated in some areas [41]. In a comprehensive meta-analysis on fourteen RCTs studies, it was shown that the eradication rate of infection with both bismuth and non-bismuth quadruple regimens was 6% higher than sequential treatment [42].

### Second-line therapy

Levofloxacin triple therapy‏ and bismuth quadruple therapy are considered as two well-known therapeutic strategies against *H. pylori* infection [43]. Levofloxacin-containing regimen contains a PPIs plus levofloxacin and amoxicillin [44]. According to the literature, eradication rate of infection in levofloxacin triple therapy and bismuth quadruple therapy is 74.5% and 78%, respectively [43, 45]. Increased resistance to quinolones has now become a major concern in reducing the clinical efficacy of levofloxacin-containing therapy; resistance to quinolones in Europe, America, and Asia is 20%, 15%, and 10% respectively [46]. Due to the adverse event rates of levofloxacin in patients, it is recommended that treatment with levofloxacin be prescribed only in cases of treatment failure [47].

### Third-line therapy

**Fig. 1:**
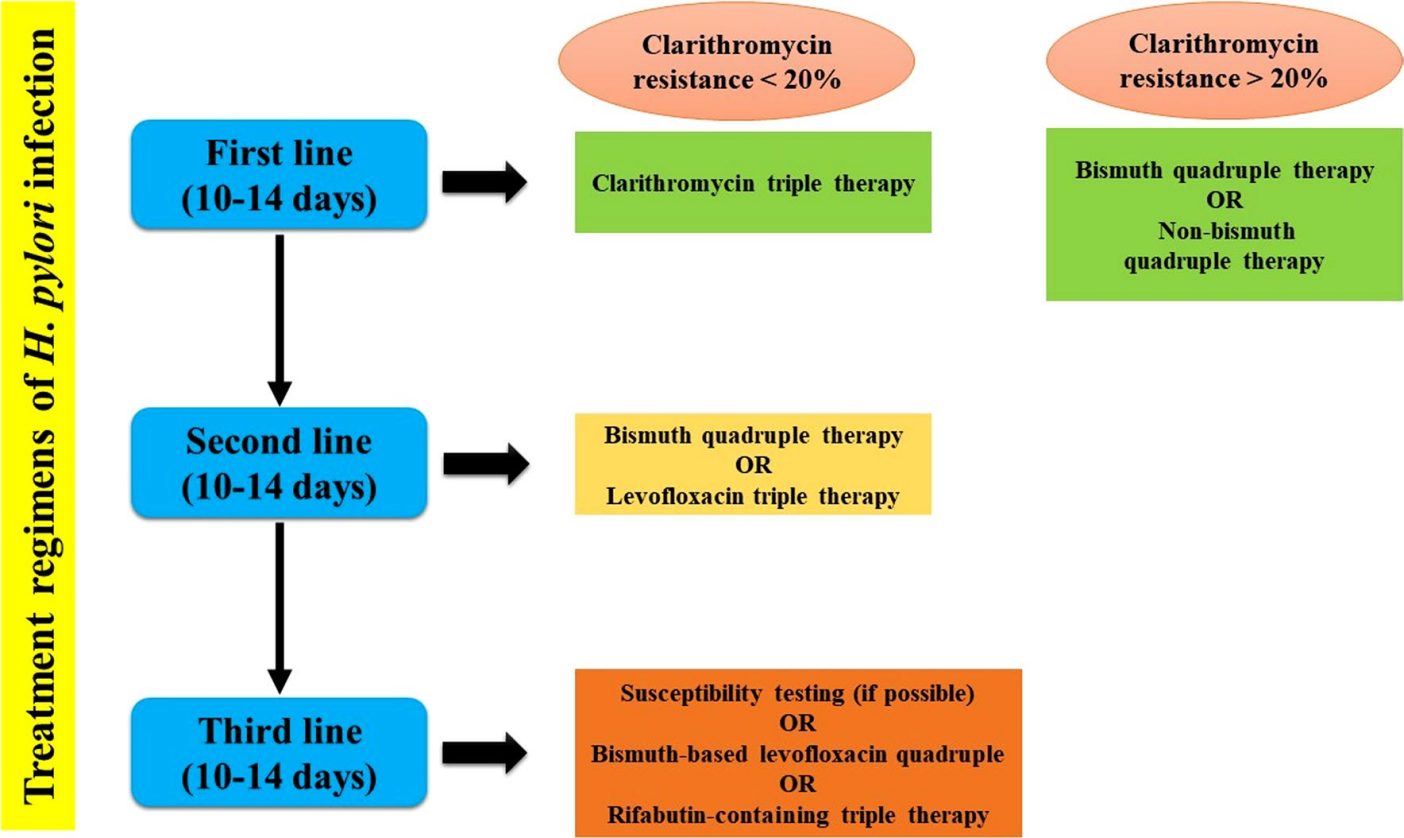
Flowchart of the three eradication regimens for the treatment of *H. pylori* infection

In general, third-line therapy is prescribed following antibiotic susceptibility testing (AST) and considered as a rescue regimen in case of failure in the first and second lines of treatment [43]. Nevertheless, due to the impossibility of testing in all areas, therefore therapeutic protocols such as bismuth-based levofloxacin quadruple therapy or rifabutin triple therapy (a PPI, rifabutin, and amoxicillin)‏ are used as ‏ alternative empiric treatments [48]. All three treatment lines are summarized in Fig. 1.

### Drawbacks of antibiotic therapy against H. pylori

Overall, there are some drawbacks versus successful antibiotic therapy that include, increasing antibiotic resistance (especially against clarithromycin and metronidazole), unfavorable acidic conditions of the stomach (degradation of antibiotics), non-FDA-approved of some antibiotics (e.g. nitazoxanide), side effects of all antibiotics, as well as toxicity and high price of some drugs [47, 49, 50]. Treatment failure may gradually lead to the progression of the primary infection to more severe complications such as peptic ulcer, MALT lymphoma, and gastric cancer [51]. In summary, probiotics help human body against *H. pylori* through direct or indirect antagonism interactions including secreting antibacterial substances (lactic acid, short-chain fatty acids, hydrogen peroxide, and bacteriocins), inhibiting bacterial colonization, enhancing mucosal barriers, and regulating the immune responses [52].

## Probiotics as anti-*H. pylori* agents

### Comprehensive definition of probiotics

Probiotics are a group of living microorganisms that generally colonize the gastrointestinal tract and have undeniable effects for improving human health [53]. Today, the clinical benefits of probiotics are widely accepted; their therapeutic applications are in disorders such as diarrhea, antibiotic-associated diarrhea, functional digestive involvements, inflammatory bowel disease, cardiovascular diseases, allergic reactions, and cancer [54]. *Lactobacillus* spp. are one of the most well-known probiotics that their anti-*H. pylori* properties have been proven [55]. According to the evidence, colonization rate of *Lactobacillus* spp. in normal human gastric is 0–10^3^ CFU (resistant to acidic conditions of the human stomach for 2 h); some *Lactobacillus* strains prevent the persistent colonization of *H. pylori* due to their specific adhesins [56]. According to the European Helicobacter Pylori Study Group (EHPSG), adjuvant therapy with probiotics can be helpful in increasing the cure rate of infections [57]. In addition to *Lactobacillus* spp., many other bacteria are accounted as bacterial probiotics against *H. pylori*; characteristics such as names of probiotics, their potential activity, in-vitro or in-vivo examinations, and country of study are listed in Table 1. However, some probiotics such as *Lactobacillus* spp. and *Bifidobacterium* spp. have been used more in clinical trials than other probiotics [58]. According to the literature, administration of a dairy product supplemented with *Lactobacillus* spp. and *Bifidobacterium* spp. increases both mucosal and systemic IgA response against to gastrointestinal infections [59]. Sheu et al. showed in their study that a yogurt containing these bacteria could improve the eradication rate of *H. pylori* infection, and also restore the depletion of *Bifidobacterium* in stool at the fifth week of treatment [60]. In addition, these bacteria can produce significant amounts of lactic acid in the stomach after successful colonization [61].

**Table 1:** List of probiotics with potential activity against *H. pylori* infection by in vitro and in vivo studies

Probiotic name	Potential activity	Human/animal/in-vitro examination	Country	Ref
*L. salivarius* WB1004	Inhibition of colonization, lactic acid	BALB/c mice	Japan	[62]
*L. acidophilus* (johnsonii) La1	Inhibition of colonization, lactic acid, H2O2, bacteriocins	Human	Switzerland	[63]
*L. johnsonii* La1	Inhibition of colonization, lactic acid, H2O2, bacteriocins	Human	Switzerland	[64]
*L. acidophilus* CRL 639	Autolysins, lactic acid	In-vitro	Sweden	[65]
*L. gasseri* OLL 2716	Anti-inflammatory activity, lactic acid	Human	Japan	[66]
*L.* reuteri	Anti-inflammatory activity (inhibition of IL-8 synthesis), lactic acid	In-vitro	Canada	[67]
*L. casei* Shirota	Biocine, lactic acid, Inhibition of colonization	Human	Netherlands	[68]
*L. casei* Shirota	Biocine, lactic acid, Inhibition of colonization	C57BL/6 mice	Greece	[69]
*L.* brevis	Arginine deiminase activity, inhibition of colonization	Human	Italy	[70]
*L. rhamnosus* R0011 and *L. acidophilus* R0052	Inhibition of colonization, lactic acid	C57BL/6 mice	Canada	[71]
*L.* salivarius	Lactic acid, bacteriocin	In-vitro	Ireland	[72]
*L. bulgaricus* BB18 and *Enterococcus faecium* MH3	Lactic acid, bulgaricin BB18, enterocin MH3	In-vitro	Bulgaria	[73]
*L. brevis* BK11 and *E. faecalis* BK61	Lactic acid, bacteriocin	In-vitro	Korea	[74]
*L. lactis* A164 and *L. lactis* BH5	Lactic acid, lacticin A164, lacticin BH5	In-vitro	Korea	[75]
*Bacillus clausii*	inhibition of colonization (bacterial cell and spores)	Human	Italy	[76]
*B. subtilis*	Amicoumacin A	In-vitro	France	[77]
Lactobacilli and Bifidobacteria	Lactic acid	Human	Germany	[78]
*Weissella confusa* PL9001	Bacteriocin, inhibition of colonization	In-vitro	Korea	[79]
*E. faecium* GM-1	Lactic acid, bacteriocin?	In-vitro	South Korea	[80]
*E. faecium* TM39	Lactic acid, bacteriocin	In-vitro	Taiwan	[81]
*Saccharomyces boulardii*	Anti-inflammatory activity	Human	Romania	[82]
*L. reuteri* ATCC 55730	Reuterin	Human	Italy	[83]
*L. rhamnosus* JB3	Antagonist of AI‐2	In-vitro	Taiwan	[84]

### Substantial mechanism of probiotics against H. pylori infection

**Fig. 2:**
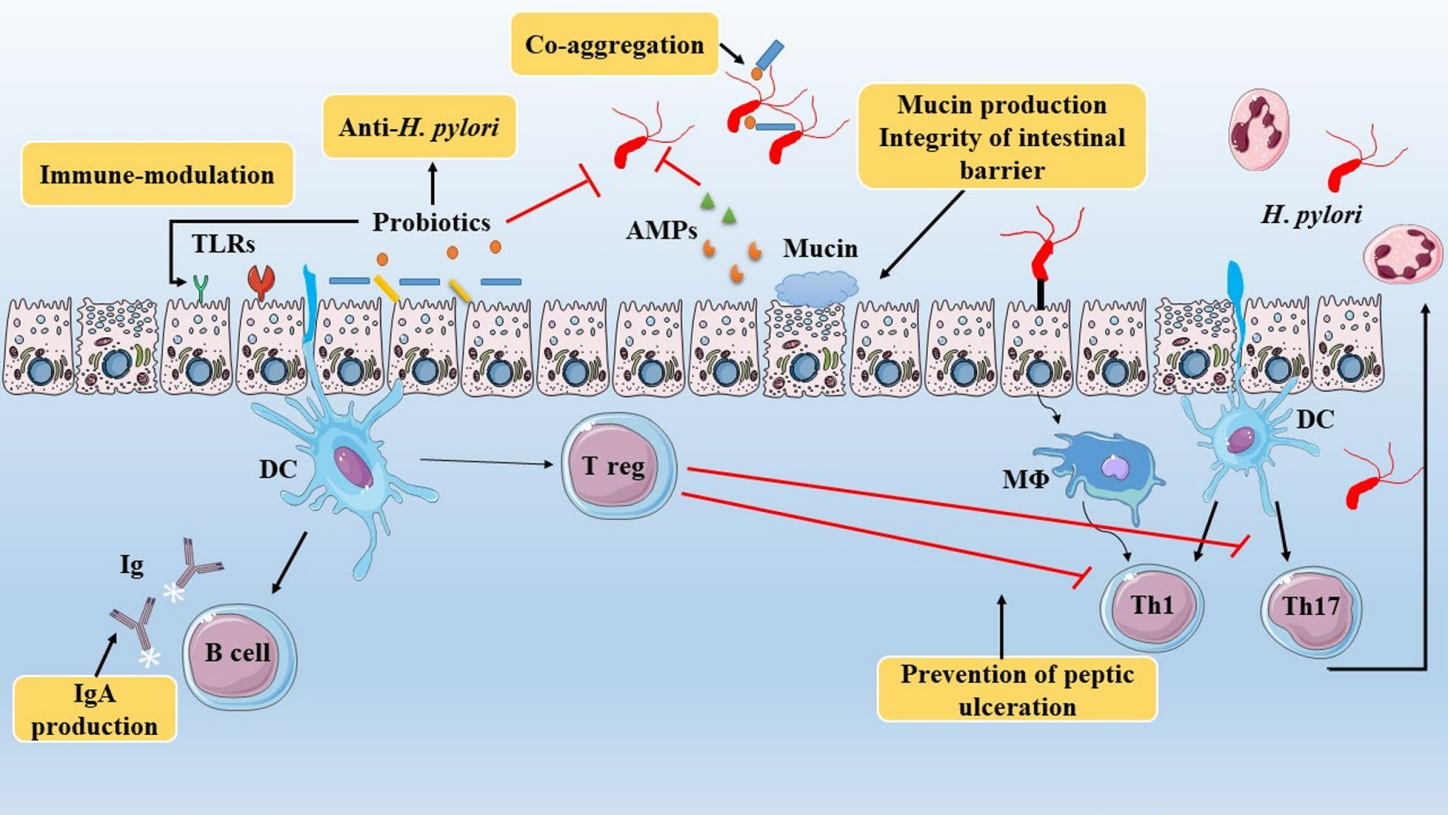
Defenses mechanisms against *H. pylori* infection which subdivided into two main mechanisms including physiological barriers and immune system. Upon entrance of *H. pylori* into the stomach, both innate and specific immunity enter the area of infection (lamina propria). Consumption of probiotics has several advantages in strengthening and stimulating immune system versus this pathogen. Antibacterial activities of probiotics direct and indirect are helpful for human health. Therapeutic effects of these bacteria in gastric tract are including immune modulation (via interaction with TLRs), anti-*H. pylori* activity, co-aggregation of invasive bacteria, decrease pH by secretion of short chain fatty acids, support epithelial barrier integrity, mucin production, as well as promoting immune cells to inhibit gastric inflammatory response particularly IL-8 production, and induction of immunoglobulin secretions

Probiotics have various mechanisms to eradicate or restrict *H. pylori* growth within the stomach of humans including, (1) inhibition the colonization of *H. pylori* via conquering gastric epithelial receptors or co-aggregation mechanism, (2) anti-*H. pylori* activity throughout the production of bacteriocins, organic acids, as well as bio-surfactants, (3) supportive role in intestinal tissues by promoting mucin synthesis, (4) modulation of immune system response, (5), induction of antigen-specific antibodies, and (6) reduction of stomach inflammation (Fig. 2). The details of each of the hypotheses proposed are discussed below.

#### Competition for binding sites

Like other bacteria, attachment is an important step in the continued colonization of *H. pylori* [85]. According to in vitro studies, *L. reuteri* inhibits the attachment of *H. pylori* via competition binding to asialo-GMI and sulfatide receptors [86]. Sakarya et al. showed that *S. boulardii* blocks the attachment of *H. pylori* to gastric epithelial cells through binding to sialic acid receptors [87]. Moreover, other probiotics such as *L. acidophilus LB*, *L. johnsonii*, *L. salivarius*, and *W. confuse* prevent the colonization of this pathogen through specific adhesion molecules [88–90]. Based on studies in thirty C57BL/6 female mice, Johenson et al. found that pre-treatment with *L. acidophilus* R0052 and *L. rhamnosus* R0011 completely inhibited the colonization of this bacterium compared to control group [71]. In addition, in a study on 13 patients infected with *H. pylori*, Myllyluoma et al. found that consuming a solution containing four probiotics for 56 days reduced the rate of infection by 27% [91].

#### Mucosal barrier

Mucous membranes are one of the first lines of defense to protect humans (or animal) against environmental pathogens; excessive secretion of mucins and large glycoproteins effectively cover the surface of gastrointestinal tracts and prevent the colonization of infectious agents, especially *H. pylori* [92]. Recent studies have shown that this bacterium inhibits the expression of several mucins genes such as MUC1 and MUC5 [93]. In vitro studies show that some probiotics e.g. *L. rhamnosus* and *L. plantarum* induce the expression of MUC2 and MUC3 genes (the most important mucins in gastrointestinal tract), leading to inhibition of *H. pylori* colonization [94]. Interestingly, Pantoflickova et al. showed in their study that consumption of *L. johnsonii* thickens the mucosal layer, which in turn prevents bacterial colonization [95].

#### Probiotics as antibiotics

Scientific studies have shown that probiotics can also act as antibiotic-producing bacteria, and are able to contain the growth of *H. pylori* by producing antimicrobial substances [96]. *Streptomyces* spp. are the largest antibiotic-producing probiotics; these bacteria produce a large number of antibiotics such as streptomycin, chloramphenicol, tetracycline, kanamycin, vancomycin, cycloserine, lincomycin, neomycin, cephalosporins, clavulanic acid [97–99]. Moreover, bacitracin as an effective antibiotic on peptidoglycan of Gram-positive bacteria is produced by *B. licheniformis* and some strains of *B. subtilis* [100].

Short-chain fatty acids produced by probiotics such as acetic acid, propionic acid, and lactic acid can lower the pH of the environment, leading to unfavorable gastric conditions for *H. pylori* [101]. Bacteriocins (antibacterial peptides) are other properties of probiotics that in turn have antagonistic activity against the survival of *H. pylori* [102]. Coconnier et al. first found that the supernatant fluid from *Lactobacillus acidophilus* LB significantly could reduce the viability of *H. pylori* [24]. In a clinical trial study, Michetti et al. showed that oral administration of culture supernatant fluid of *L. acidophilus* strain La1 had anti-*H. pylori* activity [63]. In later years, discovered that this property was due to antimicrobial nisin A [75]. Bacteriocins are a heterogeneous group of antimicrobial proteins that are mostly produced by lactic acid bacteria [103, 104]. Although studies on the effects of bacteriocin-like compounds against *H. pylori* are limited, bacteriocins with anti-*H. pylori* activity are produced by some probiotic genera such as *Pediococcus*, *Lactococcus*, *Bacillus*, *Weissella*, and *Bifidobacterium* [74, 105]. Bacteriocins reduce or inhibit the growth of *H. pylori* by a variety of mechanisms including, inducing pores in membrane, activating of autolytic enzymes, and downregulating expression of *vacA*, *cagA*, *luxS*, and *flaA* genes [52, 106–108]. In other study, Boyanova et al. introduced seven bacteriocins from *L. bulgaricus* that were able to kill both antibiotic-susceptible and-resistant bacteria [102]. However, although bacteriocins have been proposed as a new alternative to drug-resistant *H. pylori* strains, these antimicrobial peptides (AMPs) are strain-specific and are also sensitive to gastrointestinal enzymes [52, 75].

#### Co-aggregation and auto-aggregation (querish)

Co-aggregation status occurs between different species (or strains) of probiotics and pathogenic strains (heterogeneous bacteria), while in the auto-aggregation status, only species of one genus react with each other [109]. According to in vitro studies, some probiotics such as *L. reuteri* DSM17648, *L. gasseri*, and *L. johnsonni* La1 (NCC533) are able to co-aggregate with *H. pylori* strains [110, 111].

#### Immunomodulatory mechanism

Probiotics also modulate the immune system responses; Blum et al. was first showed the role of probiotics in modulating the immune system responses against *H. pylori* infection [111]. This bacterium increases the inflammatory response by promoting the secretion of TNF-α and IL-8, which in turn lead to the upregulation of gastrin-17, apoptosis, and finally peptic ulcer [91]. Yang et al. found that pre-treatment with *L. salivarius* in animal model reduced chronic gastritis through the inactivation of JAK1/STAT1 and NF-κB pathways [112]. In addition, probiotics through some processes such as upregulating the expression of MUC3, cyclooxygenase-1, and PGE2, facilitate the secretion of mucin and angiotensin, thus preventing the apoptosis of mucosal cells [113, 114].

### Probiotics as delivery system for the treatment of H. pylori infection

Although many people around the world are infected with this bacterium in the first years of life, the search for an effective vaccine began after identification of *H. pylori* by Varan and Marshall; however, the effectiveness of the vaccine is doubtful, because this bacterium suppresses the immune responses [115]. Until recently, the vaccines entered in phase III clinical trials were stopped due to insufficient immunity against this pathogen [116]. At the moment, *Lactobacillus* spp. can be used as promising candidates for oral vaccination; the most important reasons are: (1) safety, 2) being immunogenic, 3) low cost, 4) accessibility, 5) ease of administration [117]. Here are some recombinant probiotics containing *H. pylori* antigens such as *Lactococcus lactis* (UreB), *L. lactis* (NapA), *L. lactis* (CTB-UE), and *B. subtilis* (UreB); oral administration of each of them leads to an increase in serum levels of IgG and IgA [118–121].

### Probiotics and animal models

According to animal studies, researchers have shown the benefits of probiotics including, (1) elimination of *H. pylori* infection, (2) reduction of gastritis, (3) inhibition of the progression of primary infection to gastric cancer and MALT lymphoma (Table 2). According to animal experiments, probiotic supplementation can reduce the persistent colonization of *H. pylori* as well as gastric inflammation by modulating pro-inflammatory cytokines i.e. IL-8, IL-12, TNF-α, and *H. pylori*-specific IgG titer [69, 122–124]. Chronic infection can stimulate the immune system to create favorable conditions to support the growth of bacteria [125–127]. Bacterial virulence factors can disrupt the signaling pathways and cell junctions, leading to the formation of pre-cancerous lesions as hummingbird phenotype [128, 129]. Curing *H. pylori* infection is considered as the main strategy for preventing gastric MALT lymphoma and can decrease the risk of secondary gastric cancer or relapse of gastric ulcers [130, 131]. Probiotics can reduce the colonization of *H. pylori* by their protective compounds such as bacteriocins, organic acids, and biosurfactants [104]. According to the literature, *H. pylori* infection significantly affects the gastric microenvironment by several changes including DNA instability, disruption of NF-κB signaling pathway, as well as differentiation of autoreactive B cells and subsequent malignant transformation by genomic alternations [132, 133]. In general, the use of probiotics effectively modulates immune responses, reduces gastritis by reducing pro-inflammatory cytokines, and ultimately prevents *H. pylori*-induced gastric malignancies [134–136].

**Table 2:** Clinical advantages of probiotics in animal studies

Fist author	Year	Probiotic strain name	Dosage /duration	Animal model	Conclusion remarks	Ref
Ushiyama et al	2003	*L. gasseri* OLL2716	10^7^ CFU/mL	BALB/c mice	Anti-*H. pylori* effects Reduction of IL-8	[122]
Sgouras et al	2004	*L. casei* Shirota	10^8^ CFU/mL, 9 months	C57BL/6 mice	Reducing *H. pylori* colonization and decrease specific IgG titer	[69]
Henry et al	2004	*L. rhamnosus* R0011, *L. acidophilus* R0052	10^9^ CFU/mL, 9 weeks	C57BL/6 mice	Anti-*H. pylori* effects Reduce gastric inflammation	[71]
Pena et al	2005	*L. reuteri* 1602, *L. paracasei* 6798	10^9^ CFU/mL, 12 weeks	C57BL/6 mice	Reducing the TNF-α and IL-12 levels	[123]
Sgouras et al	2005	*L. johnsonii* La1 *L. amylovorus* CDE471 *L. acidophilus* IBB 801	1.5–4 × 10^8^ CFU/mL, 3 months	C57BL/6 mice	Reducing *H. pylori* colonization and decrease gastric inflammation	[137]
Brzozowski et al	2006	*L. acidophilus* R0052 *L. rhamnosus* R0011	2 × 10^9^ CFU/mL, 2 weeks	Mongolian gerbil	Reduction gastrin and gastric inflammation	[138]
Chenoll et al	2011	*B. bifidum* CECT 7366	10^9^ CFU/mL	C57BL/6 mice	Blocking colonization of *H. pylori*	[139]
Kuo et al	2013	*L. acidophilus*, *B. lactis*	5 × 10^9^ CFU/mL	Mongolian gerbil	Reduction of gastric inflammation	[140]
Kaur et al	2014	*P. acidilactici* BA28	10^9^ CFU/mL, 24 weeks	C57BL/6 mice	Anti-*H. pylori*	[141]
Kim et al	2014	*P. pentosaseus* (SL4)	10^8^ CFU/mL, 6 weeks	C57BL/6 mice	Anti-*H. pylori*	[142]
Zaman et al	2014	*L. reuteri* *L. johnsonii* *L. murinus*	10^9^ CFU/mL	Mongolian gerbil	Anti-*H. pylori*	[143]
Matsui et al	2015	*L. gasseri* SBT2055	10^9^ CFU/mL	C57BL/6 mice	Production of specific IgA, Blocking progression of MALT	[144]
Yu et al	2015	*E. faecalis* *B. longum* *L. acidophilus*	10^7^ CFU/mL	C57BL/6 mice	Reducing gastric inflammation	[145]
Pan et al	2016	*L. plantarum* ZDY 2013	10^9^ CFU/mL	C57BL/6 mice	Reducing gastric inflammation	[146]
Afsahi et al	2018	*L. plantarum* ATCC8014	10^6^ CFU/mL, 2 weeks	C57BL/6 mice	Anti-*H. pylori* Reduction of gastric inflammation	[147]
Chen et al	2018	*L. rhamnosus* JB3	5 × 10^7^ CFU/mL	C57BL/6 mice	Anti-*H. pylori* Reduction of gastric inflammation	[148]
Merino et al	2018	*L. fermentum* UCO-979C	10^7^ CFU/mL	Mongolian gerbil	Inhibited *H. pylori* SS1	[149]
Lin et al	2020	*L. fermentum* P2 (P2), *L. casei* L21 (L21), *L. rhamnosus* JB3 (JB3)	10^7^ CFU/mL	C57BL/6 mice	Reduction of gastric inflammation	[150]

### Probiotics as adjuvant therapy

#### Therapeutic effects of probiotics against H. pylori infection in children

There is ample evidence of the clinical effects of probiotics in treating and reducing bacterial load in children. Cruchet et al. conducted a randomized double-blind trial on children with asymptomatic *H. pylori* infection. In their study, the children were divided into five groups, so that four groups received probiotic *Lactobacillus* strains (live *L. paracasei* ST11 or *L. johnsonii* La1, and heat-killed *L. paracasei* ST11 or *L. johnsonii* La1),‏ and one group received placebo. They found that the C13UBT value in children receiving live *L. johnsonii* La1 was significantly lower than other groups [151]. In a similar study, asymptomatic children were randomly treated with three regimens containing standard triple therapy [8 days), *L. acidophilus* LB (daily for 8 weeks) and, *Saccharomyces boulardii* plus inulin (daily for 8 weeks). Finally, results showed that the C13UBT value was significantly lower in children receiving triple therapy and *Saccharomyces boulardii* [152]. Based on several clinical trials, it has been concluded that the rate of eradication of *H. pylori* infection increases in children receiving probiotic diets (without antibiotics). Some of these studies that suggested clinical efficacy of probiotic supplementation in the eradication of *H. pylori* infection are listed in Table 3. Based on these studies, probiotics can significantly increase *H. pylori* eradication rate particularly in patients receiving *Lactobacillus* spp. and *Bifidobacterium* spp. supplementation. These probiotics have a high potential against *H. pylori* infection using various mechanisms [55, 153]. In addition, probiotics can alter the gut microbiota to reduce gastrointestinal symptoms and drug side effects [154, 155].

**Table 3:** Available clinical trials of probiotics in the treatment of *H. pylori* infection in children

First author	Year	Type of study	Eradication therapy	Probiotic regimen	Duration	Cure rate		Statistical significance	Ref
						Case	Control		
Gotteland et al	2005	Open randomized	NA	*Saccharomyces boulardii*, *L. acidophilus*	8 weeks	12%, 6.5%	0%	*p* < 0.000	[152]
Sykora et al	2005	Double blind randomized	Omeprazole, amoxicillin, clarithromycin for 7 days	*L. casei* DN-114 001	2 weeks	84.6%	57.4%	*p* = 0.0019	[156]
Goldman et al	2006	Double blind randomized	Omeprazole, amoxicillin, clarithromycin for 7 days	*B. animalis* + *L. casei*	3 months	45.4%	37.5%	*p* < 0.01	[157]
Lionetti et al	2006	Double blind randomized	Omeprazole, amoxicillin, clarithromycin, tinidazole (sequential therapy)	*L. reuteri* ATCC 55,730	20 days	85%	80%	*p* < 0.009	[158]
Gotteland et al	2008	Double blind randomized	NA	*L. jonsonii* La1 plus cranberry, *L. jonshonii* La1, cranberry plus heat-killed *L. jonsonii* La1	3 weeks	22.9%, 14.9%, 16.9%	1.5%	*p* = 0.542	[159]
Hurduc et al	2009	Open randomized	Omeprazole, amoxicillin, clarithromycin for 7 days	*Saccharomyces boulardi*	4 weeks	93.7%	80.9%	*p* < 0.002	[82]
Szajewska et al	2009	Double blind randomized	Omeprazole, amoxicillin, clarithromycin for 7 days	*L. rhamnosus* GG	1 weeks	67.6%	68.7%	Not significant	[160]
Boonyaricaikij et al	2009	Single blind	NA	*L. gasseri* OLL2716	1 years	29.3%	6.6%	*p* = 0.03	[161]
Tolone et al	2012	NA	Omeprazole, amoxicillin, clarithromycin for 7 days	Probinul-Cadigroup	NA	88.2%	76.4%	*p* < 0.05	[162]
Zhao et al	2014	prospective randomized controlled study	Omeprazole, amoxicillin, clarithromycin for 7 days	*Saccharomyces boulardii*	7 days	85%	75.8%	*p* < 0.05	[163]
Wang et al	2014	NA	Omeprazole, amoxicillin, clarithromycin for 7 days	*L. acidophilus*, *B. bifidum*	2 weeks	83.7%	64.4%	*p* < 0.05	[164]
Akcam et al	2015	Open randomized	triple therapy (lansoprazole, amoxicillin, clarithromycin for 14 days)	*L. casei*, *L. acidophilus*, *B. lactis*	2 weeks	66.6%	68.9%	*p* = 0.78	[165]
Zhu etal	2017	Double blind randomized	Sequential, Triple therapy	Sequential-*Lactobacillus*, triple-*Lactobacillus* therapy	NA		Sequential-*Lactobacillus* and triple-*Lactobacillus* better than any of them alone (P < 0.05)	*p* < 0.01	[166]

Recently, two meta-analyses have evaluated the clinical effects of probiotics in the treatment of *H. pylori* infection in children. Li et al. evaluated data from 508 sick children; the pooled ORs for *H. pylori* eradication rate by intention-to-treat (ITT) and per-protocol (PP) analysis in children who had received probiotic supplementation and control group was 1.96 (95% CI: 1.28–3.02) and 2.25 (95% CI: 1.41–3.57), respectively [167]. In another study, Fang et al. analyzed the clinical efficacy of *Lactobacillus*-supplemented triple therapy in 484 children, and found that the relative risk (RR) of curing rate in the *Lactobacillus*-treated group was significantly higher than control group (RR: 1.19; 95% CI: 1.07–1.33); diarrhea was also significantly reduced (RR: 0.3; 95% CI: 0.10–0.85) in this group [168].

#### Therapeutic effects of probiotics against H. pylori infection in adults

In the present study we evaluated all studies conducted on the effect of probiotics against *H. pylori* infection in human (Table 4).

**Table 4:** Recent meta-analysis studies on the effect of probiotics in the treatment of *H. pylori* infection

First author	Year	Sample size	Eradication regimen	Probiotics	Conclusion remarks	Significance	Ref
Tong et al	2007	1671	First-line and second-line therapy (triple and bismuth containing quadruple therapy)	*B. clausii*, *Lactobacillus*, *Saccharomyces*	ER: RR: 1.84; 95% CI: 1.34–2.54 AE: 0.44; 95% CI: 0.3–0.6	Both significant AE was adverse event ER was eradication rate	[169]
Sachdeva et al	2009	963	First-line therapy (Triple and Quadruple)	*Lactobacillus*, *Bifidobacterium*	ER: 1.91; 95% CI: 1.3–2.6 AE: 0.51; 95% CI: 0.1–2.5 but AE was not significant	Reduction of adverse event rate was not significant	[170]
Zou et al	2009	1372	First-line therapy (Triple)	*Lactobacillus*	ER: 1.78; 95% CI: 1.21–2.62 AE: OR was 0.49 (95% CI = 0.24–1.02)	Both significant	[171]
Szajewska et al	2010	1307	First-line therapy (Triple)	*S. boulardii*	ER: 1.13, 95% CI 1.05–1.21 AE: RR 0.46, 95% CI 0.3–0.7	Both significant	[172]
Zheng et al	2013	1163	First-line therapy (Triple)	*Lactobacillus*	RR: 1.14; 95% CI: 1.06–1.22 (significant increase of eradication rate) but no significant reduction of overall adverse event	Reduction of adverse event rate was not significant	[173]
Wang et al	2013	1469	First-line and second-line therapy (triple and bismuth containing quadruple therapy)	*Bifidobacterium*, *Lactobacillus*	ER: 2.066 (95% CI, 1.398–3.055 AE: 0.305; 95% CI, 0.117–0.793)	Both significant	[174]
Zhu et al	2014	2259	standard triple H. pylori	*Lactobacillus*, *Bifidobacterium*, *Saccharomyces*	ER: 1.67 (95%CI: 1.38–2.02) AE: (OR = 0.49, 95%CI: 0.26–0.94	Both significant	[175]
Dang et al	2014	4459	First-line therapy (Triple)	*L. acidophilus*, *L. casei* DN-114001, *L. gasseri*, *Bifidobacterium infantis* 2036	Curing rate was significantly increase in probiotics (RR: 1.11; 95%CI: 1–1.1) as well as reduce of adverse event (RR: 0.73; 95%CI: 0.5–0.9)	Both significant	[176]
Zhang et al	2015	6997	First-line and second-line therapy (triple And bismuth containing quadruple therapy)	*Lactobacillus*, *Bifidobacterium*, *Streptococcus*, *Saccharomyces*, *Enterococcus*, *Bacillus*	ER: RR = 1.13; 95%CI: 1.10–1.16 AE: RR = 0.59; 95%CI: 0.48–0.71	Both significant	[177]
Lu et al	2016	3349	First-line therapy (triple)	*Lactobacilli*, *Bifidobacteria*, *Bacillus clausii*, *E. faecium*	ER: OR 1.44, 95% CI: 0.87, 2.39 but not significant AE: probiotics did improve the adverse effects OR 0.56, 95% CI: 0.31, 1.01	Both not significant	[178]
Lu et al	2016	2306	First-line therapy (Triple)	*Lactobacillus*, *Bifidobacterium*	Eradication rate in probiotic supplementation group was significantly higher than control (RR: 1.15; 95%CI: 1.1–1.2) and reducing adverse event (RR: 0.71; 0.5–0.9) probiotic supplementation increased eradication of triple therapy in both 7 and 14-days	Both significant	[179]
Si et al	2017	2466	First-line therapy (bismuth containing quadruple therapy)	*Lactobacillus*	Eradication rate was significant increase in probiotics (89% vs. 84.7% for first-line) (91% vs. 73.8% for second-line)	significant	[180]
Losurdo et al	2018	NA	NA	*Lactobacillus*	ER: UBT value: 8.61% vs. 0.19% AE: 1, 95%CI: 0.06–18.08 not siginificANT FOR AE	Reduction of adverse event rate was not significant	[181]
Shi et al	2019	8924	First-line therapy (Triple and Quadruple)	*Lactobacillus*	RR: 1.14; 95%CI: 1.10–1.18 (significant increase of eradication rate) and reduced side effects	Both significant	[182]
Yu et al	2019	724	First-line therapy (Triple	*Lactobacillus*	Eradication rate was significantly increase in Lactobacillus supplement group (RR: 1.1; 95%CI: 1–1.2) and derease significantly adverse event (RR: 0.36; 95%CI: 0.1–0.7)	Both significant	[183]
Pourmasoumi et al	2019	525	First-line therapy (Triple and Quadruple)	*Lactobacillus*, *Bifidobacterium Saccharomyces*	Eradication: RR: 1.28; 95% CI: 1.15–1.43 Adverse: RR: 0.90; 95% CI: 0.69–1.16	Both significant	[184]
Zhou et al	2019	3592	First-line therapy (Triple)	*S. boulardii*	ER: 1.09, 95% CI:1.05‐1.13 AE: RR = 0.33, 95%CI:0.16‐0.69	Both significant	[185]

According to the literature, probiotic supplementation increases the rate of infection eradication during first- and second-line treatment (Table 4). However, according to some studies, probiotic supplementation was significantly ineffective in improving the eradication rate of infection; in their network meta-analysis, Wang et al. found that probiotics in combination with triple therapy could not increase the eradication rate of infection [186]. In addition, most studies have shown that adverse events were significantly lower in the group receiving probiotics plus antibiotic than in the control group, but this was not the case in a number of other studies [178, 181, 185]. It is important to note that probiotics alone are not effective, but can only be prescribed as adjunctive therapy in clinical improvement [174]. In recent, using data of 467 patients with treatment failure, we showed that *Lactobacillus*-containing bismuth quadruple therapy for 10 days, significantly increases the cure rate of *H. pylori* infection in patients with previous treatment failure (RR: 1.77; 95% CI: 1.11–2.83; *p* value: 0.01. (Among all probiotics, the clinical effects of *Lactobacillus* spp. and *S. boulardii* have been further studied; *S.* boulardii and *Lactobacillus* species such as *L. casei*, *L. reuteri*, and *L. rhamnosus* GG are all safe and improve the quality of treatment [172, 183, 185]. It seems that multi-strain probiotics supplementation has a significant effect on the treatment of infection [173, 181, 182]. In accordance with this theory, Lu et al. showed that multi-strain probiotics (*Bacillus*, *Saccharomyces*, *Streptococcus*, *Bifidobacterium*, and *Lactococcus*) significantly increased the eradication rate of infection (RR: 1.12; 95% CI: 1.07–1.18; *p* value: 0.00001); however, heterogeneity was significant in their study [179]. In general, according to various studies, probiotic supplements are considered as a reliable strategy to increase the quality of treatment in individuals with treatment-naïve or treatment-failure.

#### Use of probiotics in the prevention of H. pylori infection

Vaccine prophylaxis as a suitable strategy has become a big challenge for this bacterium, because in many people it is colonized in childhood, the rate of infection is high, as well as the immunology of the stomach is unclear [187]. According to the results of a cohort study on 308 *H. pylori*-negative children, it was defined that the infection rate in groups receiving *L. gasseri* OLL2716 (LG21) was less than control group (4.1% vs 8.1%, respectively); nevertheless; the results was not significant [161].

#### Diversity of gut microbiota during H. pylori treatment with probiotic supplementation

In total, about 100 trillion bacteria have been colonized in the human body. Gastrointestinal microflora is one of the most complex microbial ecosystem, and protects host against colonization of pathogenic microorganisms [188, 189]. Imbalance in this ecosystem due to the excessive use of antibiotics leads to several disorders such as inflammatory bowel disease (IBD), metabolic syndrome and even colon cancer [190–192]. According to the literature, *H. pylori* infection can cause dysbiosis in the intestinal microbiota, but short- and long-term changes in human gut microbiome after *H. pylori* infection are controversial [193, 194]. In their meta-analysis, Ye et al. showed that the during long-term follow-up the frequency of *Actinobacteria* and *Bacteroidetes* was reduced; they also found that the frequency of *Enterococcus* and *Enterobacteriaceae* was increased, while Proteobacteria after a short-term increase, again returned to their normal amounts during long-term follow-up [194]. There is limit information about the effects of probiotics on gut microbiota during the *H. pylori* infection. In their study, Oh et al. evaluated functional changes in intestinal microbiota using the Illumina MiSeq system after standard anti-*H. pylori* treatment and probiotic supplementation. They found that the expression of genes involved in selenocompound metabolism pathway was significantly reduced in patients receiving probiotic; this phenomenon can be led to a reduction in side effects such as intestinal irritation as well as antibiotic resistance [195]. Wang et al., recently explored the effect of anti-*H. pylori* concomitant therapy *vs*. concomitant therapy plus probiotic supplementation (with *S. boulardii*) on the alternation of gut and throat microbiota in human subjects. They showed that there was significant quantitative and qualitative alternations in microbiota composition in both concomitant anti-*H. pylori* therapy and concomitant therapy plus probiotic supplementation groups. Nevertheless, in probiotic supplementation group most changes in gut microbiota reverted after 71 days (except for *Bacteroides* spp. and yeast counts), whereas changes in the throat microbiota were persistent. In addition, antibiotic resistance rate of bacteria such as *Enterobacteriaceae*, *Enterococcus* spp., and *Bacteroides* spp. was significantly higher in patients receiving concomitant therapy than patients receiving concomitant therapy plus probiotic supplementation. Moreover, their study revealed that co-administration of probiotics in the treatment of *H. pylori* infection could be more effective than post-antibiotic supplementation [196]. In a recent study by Cárdenas et al. the clinical effects of *S. boulardii* CNCM I-745 on gut microbiota of patients receiving standard anti-*H. pylori* therapy was evaluated. According to their results, supplementation with this probiotic significantly reduced gastrointestinal symptoms (*p* = 0.028); alterations in gut microbiota was also seen with higher abundance of Enterobacteria and lower abundance of Bacteroides and Clostridia upon treatment completion (*p* = 0.0156) [197]. In general, the antimicrobial activity of probiotics kills or inhibits the growth of resistant bacteria and ultimately reduces antibiotic resistance [195, 196]. According to information at https://clinicaltrials.gov/, all clinical trial studies on the effects of probiotic supplements on the eradication of *H. pylori* by August 2021 are listed in Table 5.

**Table 5:** Clinical trials on the role of probiotics in treating *H. pylori* infections (https://clinicaltrials.gov/)

Row	Identifier	Start year	Participants	Allocation	Intervention model	Masking	Primary Purpose	Status	Country
1	NCT04319991	2019	100	Randomized	Parallel assignment	Single (Participant)	Supportive Care	Recruiting	Taiwan
2	NCT01115296	2010	100	Randomized	Parallel assignment	Quadruple (Participant, Care Provider, Investigator, Outcomes Assessor)	Treatment	Unknown	Italy
3	NCT03150394	2017	80	Randomized	Parallel assignment	Double (Participant, Investigator)	Treatment	Unknown	Spain
4	NCT04178187	2019	800	Randomized	Parallel assignment	Single (Participant)	Treatment	Recruiting	Greece
5	NCT01969331	2008	804	Randomized	Parallel assignment	Triple (Participant, Care Provider, Investigator)	Treatment	Completed	Croatia
6	NCT02645201	2016	0	Randomized	Parallel assignment	Triple (Participant, Care Provider, Investigator)	Treatment	Withdrawn	Belgium, Croatia, Germany, Israel, Slovenia
7	NCT03220542	2016	360	Randomized	Factorial assignment	Single (Participant)	Treatment	Unknown	Korea
8	NCT03722433	2018	200	Randomized	Parallel assignment	Double (Participant, Care Provider)	Treatment	Unknown	Taiwan
9	NCT03997279	2019	200	Randomized	Parallel assignment	Triple (Participant, Care Provider, Investigator)	Treatment	Unknown	Sebria
10	NCT03377933	2019	40	N/A	Single group assignment	None (Open Label)	Treatment	Unknown	China
11	NCT04473079	2020	100	Randomized	Parallel assignment	Quadruple (Participant, Care Provider, Investigator, Outcomes Assessor)	Supportive Care	Recruiting	Thailand
12	NCT04527055	2020	252	Randomized	Parallel Assignment	Single (Outcomes Assessor)	Treatment	Enrolling by invitation	Taiwan
13	NCT03297242	2017	30	N/A	N/A	N/A	N/A	Unknown	China
14	NCT04786938	2016	63	Randomized	Parallel assignment	Single (Participant)	Treatment	Completed	Ecuador
15	NCT02689583	2016	3000	Randomized	Parallel assignment	Single (Participant)	Treatment	Unknown	China
16	NCT03688828	2018	776	Randomized	Parallel assignment	Triple (Participant, Investigator, Outcomes Assessor)	Treatment	Recruiting	China
17	NCT03404440	2016	56	Randomized	Parallel assignment	Double (Participant, Investigator)	Treatment	Completed	Italy
18	NCT01456728	2011	56	Randomized	Parallel assignment	Double (Participant, Investigator)	Treatment	Completed	Bulgaria
19	NCT02051348	2014	24	Non-Randomized	Crossover assignment	Single (Participant)	Treatment	Completed	Ireland

## Disadvantages and limitations

Despite extensive research on the effectiveness of probiotics in eradicating *H. pylori* infection, there are many challenges in this filed. Due to differences in study design, duration of treatment, and variety of probiotics between clinical trial studies, there is no a reliable homogeneity between them, which in turn affects the interpretation of results. In addition, due to the small sample size of studies, more research needs to be done with larger populations. Unfortunately, in some studies, there is no significant difference between the probiotic supplement group and the control group. Finally, although the exact role of probiotics in the prevention or treatment of *H. pylori* remains unknown, consumption of probiotics may be associated with side effects such as increasing in serum histamine and also digestive disorders [198].

## Conclusions and future perspectives

*H. pylori* is one of the most successful pathogens in the gastrointestinal tract, which through its virulence factors creates a complex interaction with the human host. Chronic infection caused by this bacterium leads to severe clinical outcomes. The frequency with this bacterium is high in developing countries and poor socio-economic conditions, so that people living in these conditions are generally at high risk for re-infection. Moreover, self-medication with antibiotics on the one hand, and the spread of resistant strains on the other hand, all are considered as a serious threat for the successful eradication of this bacterium. Over the decades, the controversial results of all conducted studies about the treatment of *H. pylori* infection have been led to the failure to the eradication of this pathogen. Hence, probiotics have been considered by many researchers around the world. In the present study, based on in vitro, animal studies, and human clinical trials, we demonstrated the beneficial effects of probiotics against *H. pylori* infection. However, those alone are not effective in treating the bacterial infection. In addition, the anti-*H. pylori* activity of probiotics is strain-specific and remains as a mysterious phenomenon. To date, the therapeutic effects of probiotics against resistant strains of the bacterium have not been evaluated, and whole genome sequencing may solve the existing puzzles. It seems that to decrease the heterogeneity of results and make better decisions, future studies should focus on items such as genus/species, dosage, formulation, and treatment course.

## Data Availability

All data generated or analyzed during this study are included in this published article.

## References

[1] Karbalaei M, Khorshidi M, Sisakht-pour B, Ghazvini K, Farsiani H, Youssefi M (2020). What are the effects of IL-1β (rs1143634), IL-17A promoter (rs2275913) and TLR4 (rs4986790) gene polymorphism on the outcomes of infection with H. pylori within as Iranian population; A systematic review and meta-analysis. Gene Rep..

[2] Hooi JK, Lai WY, Ng WK, Suen MM, Underwood FE, Tanyingoh D (2017). Global prevalence of Helicobacter pylori infection: systematic review and meta-analysis. Gastroenterology.

[3] Keikha M (2020). Is there a relationship between Helicobacter pylori vacA i1 or i2 alleles and development into peptic ulcer and gastric cancer? A meta-analysis study on an Iranian population. New Microbes New Infect..

[4] Youssefi M, Tafaghodi M, Farsiani H, Ghazvini K, Keikha M (2021). Helicobacter pylori infection and autoimmune diseases; Is there an association with systemic lupus erythematosus, rheumatoid arthritis, autoimmune atrophy gastritis and autoimmune pancreatitis? A systematic review and meta-analysis study. J Microbiol Immunol Infect..

[5] Gravina AG, Zagari RM, De Musis C, Romano L, Loguercio C, Romano M (2018). Helicobacter pylori and extragastric diseases: a review. World J Gastroenterol.

[6] O’connor A, O’morain CA, Ford AC (2017). Population screening and treatment of Helicobacter pylori infection. Nat Rev Gastroenterol Hepatol.

[7] Hildebrand P, Bardhan P, Rossi L, Parvin S, Rahman A, Arefin MS (2001). Recrudescence and reinfection with Helicobacter pylori after eradication therapy in Bangladeshi adults. Gastroenterology.

[8] Keikha M (2020). The association between Helicobacter pylori eradication in peptic ulcer patients and gastric cancer? Investigation in an East-Asian population. Trends Pharm Sci..

[9] Lee Y-C, Chiang T-H, Chou C-K, Tu Y-K, Liao W-C, Wu M-S (2016). Association between Helicobacter pylori eradication and gastric cancer incidence: a systematic review and meta-analysis. Gastroenterology.

[10] Georgopoulos S, Papastergiou V (2020). An update on current and advancing pharmacotherapy options for the treatment of H. pylori infection. Expert Opin Pharmacother..

[11] Savoldi A, Carrara E, Graham DY, Conti M, Tacconelli E (2018). Prevalence of antibiotic resistance in Helicobacter pylori: a systematic review and meta-analysis in World Health Organization regions. Gastroenterology.

[12] Graham DY, Lu H, Shiotani A (2017). Failure of optimized dual proton pump inhibitor amoxicillin therapy: what now?. Saudi J Gastroenterol.

[13] Thung I, Aramin H, Vavinskaya V, Gupta S, Park J, Crowe S (2016). the global emergence of Helicobacter pylori antibiotic resistance. Aliment Pharmacol Ther.

[14] Hu Y, Zhang M, Lu B, Dai J (2016). Helicobacter pylori and antibiotic resistance, a continuing and intractable problem. Helicobacter.

[15] Goderska K, Pena SA, Alarcon T (2018). Helicobacter pylori treatment: antibiotics or probiotics. Appl Microbiol Biotechnol.

[16] Malfertheiner P, Megraud F, O’Morain C, Bazzoli F, El-Omar E, Graham D (2007). Current concepts in the management of Helicobacter pylori infection: the Maastricht III Consensus Report. Gut.

[17] De Francesco V, Zullo A, Ierardi E, Vaira D (2009). Minimal inhibitory concentration (MIC) values and different point mutations in the 23S rRNA gene for clarithromycin resistance in Helicobacter pylori. Dig Liver Dis.

[18] Guevara B, Cogdill AG (2020). Helicobacter pylori: a review of current diagnostic and management strategies. Dig Dis Sci.

[19] Graham DY, Lu H, Yamaoka Y. A report card to grade Helicobacter pylori therapy. Wiley Online Library; 2007. 10.1111/j.1523-5378.2007.00518.x 17669098

[20] Gong EJ, Yun S-C, Jung H-Y, Lim H, Choi K-S, Ahn JY (2014). Meta-analysis of first-line triple therapy for helicobacter pylori eradication in Korea: is it time to change?. J Korean Med Sci.

[21] Eslami M, Bahar A, Keikha M, Karbalaei M, Kobyliak N, Yousefi B (2020). Probiotics function and modulation of the immune system in allergic diseases. Allergologia et Immunopathologia..

[22] Eslami M, Sadrifar S, Karbalaei M, Keikha M, Kobyliak NM, Yousefi B (2019). Importance of the microbiota inhibitory mechanism on the Warburg effect in colorectal cancer cells. J Gastrointest Cancer..

[23] Collado MC, Isolauri E, Salminen S, Sanz Y (2009). The impact of probiotic on gut health. Curr Drug Metab.

[24] Coconnier M-H, Lievin V, Hemery E, Servin AL (1998). Antagonistic activity against Helicobacter infection in vitro and in vivo by the human Lactobacillus acidophilus strain LB. Appl Environ Microbiol.

[25] García A, Sáez K, Delgado C, González CL (2012). Low co-existence rates of Lactobacillus spp. and Helicobacter pylori detected in gastric biopsies from patients with gastrointestinal symptoms. Rev Esp Enferm Dig.

[26] Eslami M, Yousefi B, Kokhaei P, Moghadas AJ, Moghadam BS, Arabkari V (2019). Are probiotics useful for therapy of Helicobacter pylori diseases?. Comp Immunol Microbiol Infect Dis.

[27] Lesbros-Pantoflickova D, Corthesy-Theulaz I, Blum AL (2007). Helicobacter pylori and probiotics. J Nutr.

[28] Malfertheiner P, Megraud F, O'morain C, Gisbert J, Kuipers E, Axon A, et al. Management of Helicobacter pylori infection—the Maastricht V/Florence consensus report. Gut. 2017;66(1):6–30. 10.1136/gutjnl-2016-312288 27707777

[29] Paz S, Lasa J, Zubiaurre I (2020). Helicobacter pylori infection. Frequency of first-line treatment failure Medicina.

[30] Kim TH, Park JM, Cheung DY, Oh JH (2020). Comparison of 7-and 14-day eradication therapy for Helicobacter pylori with first-and second-line regimen: randomized clinical trial. J Korean Med Sci..

[31] Elitsur Y, Lawrence Z, Rüssmann H, Koletzko S (2006). Primary clarithromycin resistance to Helicobacter pylori and therapy failure in children: the experience in West Virginia. J Pediatr Gastroenterol Nutr.

[32] Horiki N, Omata F, Uemura M, Suzuki S, Ishii N, Fukuda K (2012). Risk for local recurrence of early gastric cancer treated with piecemeal endoscopic mucosal resection during a 10-year follow-up period. Surg Endosc.

[33] Koletzko S, Richy F, Bontems P, Crone J, Kalach N, Monteiro ML (2006). Prospective multicentre study on antibiotic resistance of Helicobacter pylori strains obtained from children living in Europe. Gut.

[34] Ogata SK, Godoy APO, da Silva Patricio FR, Kawakami E (2013). High Helicobacter pylori resistance to metronidazole and clarithromycin in Brazilian children and adolescents. J Pediatr Gastroenterol Nutr.

[35] De Francesco V, Giorgio F, Hassan C, Manes G, Vannella L, Panella C, et al. Worldwide H. pylori antibiotic resistance: a systematic. J Gastrointestin Liver Dis. 2010;19(4):409–14. 21188333

[36] Falsafi T, Mobasheri F, Nariman F, Najafi M (2004). Susceptibilities to different antibiotics of Helicobacter pylori strains isolated from patients at the pediatric medical center of Tehran. Iran J Clin Microbiol.

[37] Yao C-C, Kuo C-M, Hsu C-N, Yang S-C, Wu C-K, Tai W-C (2019). First-line Helicobacter pylori eradication rates are significantly lower in patients with than those without type 2 diabetes mellitus. Infect Drug Resist.

[38] Malfertheiner P, MEÂGRAUD F, O'morain C, Hungin A, Jones R, Axon A. Current concepts in the management of Helicobacter pylori infection-The Maastricht 2-2000 Consensus Report. Aliment Pharmacol Ther. 2002;16:167–80. 10.1046/j.1365-2036.2002.01169.x 11860399

[39] Liou J-M, Fang Y-J, Chen C-C, Bair M-J, Chang C-Y, Lee Y-C (2016). Concomitant, bismuth quadruple, and 14-day triple therapy in the first-line treatment of Helicobacter pylori: a multicentre, open-label, randomised trial. The Lancet.

[40] Luther J, Higgins PD, Schoenfeld PS, Moayyedi P, Vakil N, Chey WD (2010). Empiric quadruple vs. triple therapy for primary treatment ofhelicobacter pylori infection: systematic review and meta-analysis of efficacy and tolerability. Am J Gastroenterol.

[41] Perri F, Festa V, Merla A, Quitadamo M, Clemente R, Andriulli A (2002). Amoxicillin-tetracycline combinations are inadequate as alternative therapies for helicobacter pylori infection. Helicobacter.

[42] Fallone CA, Chiba N, van Zanten SV, Fischbach L, Gisbert JP, Hunt RH (2016). The Toronto consensus for the treatment of Helicobacter pylori infection in adults. Gastroenterology.

[43] Chey WD, Leontiadis GI, Howden CW, Moss SF (2017). ACG clinical guideline: treatment of Helicobacter pylori infection. Off J Am Coll Gastroenterol ACG.

[44] Zagari RM, Rabitti S, Eusebi LH, Bazzoli F (2018). Treatment of Helicobacter pylori infection: a clinical practice update. Eur J Clin Investig.

[45] Chen PY, Wu MS, Chen CY, Bair MJ, Chou CK, Lin JT (2016). Systematic review with meta-analysis: the efficacy of levofloxacin triple therapy as the first-or second-line treatments of Helicobacter pylori infection. Aliment Pharmacol Ther.

[46] Liang C-M, Cheng J-W, Kuo C-M, Chang K-C, Wu K-L, Tai W-C (2014). Levofloxacin-containing second-line anti-Helicobacter pylori eradication in Taiwanese real-world practice. Biomed J.

[47] Antos D, Schneider-Brachert W, Bästlein E, Hänel C, Haferland C, Buchner M (2006). 7-day triple therapy of Helicobacter pylori infection with levofloxacin, amoxicillin, and high-dose esomeprazole in patients with known antimicrobial sensitivity. Helicobacter.

[48] Gisbert J, Calvet X (2012). rifabutin in the treatment of refractory Helicobacter pylori infection. Aliment Pharmacol Ther.

[49] Flores-Treviño S, Mendoza-Olazarán S, Bocanegra-Ibarias P, Maldonado-Garza HJ, Garza-González E (2018). Helicobacter pylori drug resistance: therapy changes and challenges. Expert Rev Gastroenterol Hepatol.

[50] Iqbal U, Khara HS, Akhtar D, Hu Y, Anwar H, Haq KF (2020). Safety and efficacy of Nitazoxanide-based regimen for the eradication of helicobacter pylori infection: a systematic review and meta-analysis. Gastroenterol Res.

[51] Karbalaei M, Keikha M (2021). Rescue effects of Lactobacillus-containing bismuth regimens after Helicobacter pylori treatment failure. New Microbes New Infect.

[52] Ji J, Yang H (2020). Using probiotics as supplementation for Helicobacter pylori antibiotic therapy. Int J Mol Sci.

[53] Sazawal S, Dhingra U, Hiremath G, Sarkar A, Dhingra P, Dutta A (2010). Prebiotic and probiotic fortified milk in prevention of morbidities among children: community-based, randomized, double-blind, controlled trial. PLoS ONE.

[54] Yousefi B, Eslami M, Ghasemian A, Kokhaei P, Sadeghnejhad A (2019). Probiotics can really cure an autoimmune disease?. Gene Rep.

[55] Gotteland M, Brunser O, Cruchet S (2006). Systematic review: are probiotics useful in controlling gastric colonization by Helicobacter pylori?. Aliment Pharmacol Ther.

[56] Gupta V, Garg R (2009). Probiotics. Indian J Med Microbiol.

[57] Malfertheiner P, Megraud F, O'Morain CA, Atherton J, Axon AT, Bazzoli F (2012). Management of Helicobacter pylori infection—the Maastricht IV/Florence consensus report. Gut.

[58] Ruggiero P (2014). Use of probiotics in the fight against Helicobacter pylori. World J Gastrointest Pathophysiol.

[59] Wang K-Y, Li S-N, Liu C-S, Perng D-S, Su Y-C, Wu D-C (2004). Effects of ingesting Lactobacillus-and Bifidobacterium-containing yogurt in subjects with colonized Helicobacter pylori. Am J Clin Nutr.

[60] Sheu BS, Wu JJ, Lo CY, Wu HW, Chen JH, Lin YS (2002). Impact of supplement with Lactobacillus-and Bifidobacterium-containing yogurt on triple therapy for Helicobacter pylori eradication. Aliment Pharmacol Ther.

[61] Chatterjee S, Kar P, Das T, Ray S, Gangulyt S, Rajendiran C (2013). Randomised placebo-controlled double blind multicentric trial on efficacy and safety of Lactobacillus acidophilus LA-5 and Bifidobacterium BB-12 for prevention of antibiotic-associated diarrhoea. J Assoc Phys India.

[62] Kabir A, Aiba Y, Takagi A, Kamiya S, Miwa T, Koga Y (1997). Prevention of Helicobacter pylori infection by lactobacilli in a gnotobiotic murine model. Gut.

[63] Michetti P, Dorta G, Wiesel P, Brassart D, Verdu E, Herranz M (1999). Effect of whey-based culture supernatant of Lactobacillus acidophilus (johnsonii) La1 on Helicobacter pylori infection in humans. Digestion.

[64] Felley CP, Corthésy-Theulaz I, Rivero J-LB, Sipponen P, Kaufmann M, Bauerfeind P, et al. Favourable effect of an acidified milk (LC-1) on Helicobacter pylori gastritis in man. Eur J Gastroenterol Hepatol. 2001;13(1):25–9. 10.1097/00042737-200101000-00005 11204805

[65] Lorca GL, Wadström T, De Valdez GF, Ljungh Å (2001). Lactobacillus acidophilus autolysins inhibit Helicobacter pylori in vitro. Curr Microbiol.

[66] Sakamoto I, Igarashi M, Kimura K, Takagi A, Miwa T, Koga Y (2001). Suppressive effect of Lactobacillus gasseri OLL 2716 (LG21) on Helicobacter pylori infection in humans. J Antimicrob Chemother.

[67] Ma D, Forsythe P, Bienenstock J (2004). Live Lactobacillus reuteri is essential for the inhibitory effect on tumor necrosis factor alpha-induced interleukin-8 expression. Infect Immun.

[68] Cats A, Kuipers E, Bosschaert M, Pot R, Vandenbroucke-Grauls C, Kusters J (2003). Effect of frequent consumption of a Lactobacillus casei-containing milk drink in Helicobacter pylori-colonized subjects. Aliment Pharmacol Ther.

[69] Sgouras D, Maragkoudakis P, Petraki K, Martinez-Gonzalez B, Eriotou E, Michopoulos S (2004). In vitro and in vivo inhibition of Helicobacter pylori by Lactobacillus casei strain Shirota. Appl Environ Microbiol.

[70] Linsalata M, Russo F, Berloco P, Caruso ML, Matteo GD, Cifone MG (2004). The influence of Lactobacillus brevis on ornithine decarboxylase activity and polyamine profiles in Helicobacter pylori-infected gastric mucosa. Helicobacter.

[71] Johnson-Henry KC, Mitchell DJ, Avitzur Y, Galindo-Mata E, Jones NL, Sherman PM (2004). Probiotics reduce bacterial colonization and gastric inflammation in H. pylori-infected mice. Dig Dis Sci.

[72] Ryan KA, Daly P, Li Y, Hooton C, O'Toole PW (2008). Strain-specific inhibition of Helicobacter pylori by Lactobacillus salivarius and other lactobacilli. J Antimicrob Chemother.

[73] Simova E, Beshkova D, Dimitrov ZP (2009). Characterization and antimicrobial spectrum of bacteriocins produced by lactic acid bacteria isolated from traditional Bulgarian dairy products. J Appl Microbiol.

[74] Lim E-S (2015). Purification and characterization of two bacteriocins from Lactobacillus brevis BK11 and Enterococcus faecalis BK61 showing anti-Helicobacter pylori activity. J Korean Soc Appl Biol Chem.

[75] Kim T-S, Hur J-W, Yu M-A, Cheigh C-I, Kim K-N, Hwang J-K (2003). Antagonism of Helicobacter pylori by bacteriocins of lactic acid bacteria. J Food Prot.

[76] Nista EC, Candelli M, Cremonini F, Cazzato IA, Zocco MA, Franceschi F (2004). Bacillus clausii therapy to reduce side-effects of anti-Helicobacter pylori treatment: randomized, double-blind, placebo controlled trial. Aliment Pharmacol Ther.

[77] Pinchuk IV, Bressollier P, Verneuil B, Fenet B, Sorokulova IB, Mégraud F (2001). In vitro anti-helicobacter pyloriactivity of the probiotic strain bacillus subtilis 3 is due to secretion of antibiotics. Antimicrob Agents Chemother.

[78] de Vrese M, Kristen H, Rautenberg P, Laue C, Schrezenmeir J (2011). Probiotic lactobacilli and bifidobacteria in a fermented milk product with added fruit preparation reduce antibiotic associated diarrhea and Helicobacter pylori activity. J Dairy Res.

[79] Nam H, Ha M, Bae O, Lee Y (2002). Effect of Weissella confusa strain PL9001 on the adherence and growth of Helicobacter pylori. Appl Environ Microbiol.

[80] Kang J, Lee M (2005). In vitro inhibition of Helicobacter pylori by Enterococcus faecium GM-1. Can J Microbiol.

[81] Tsai C-C, Huang L-F, Lin C-C, Tsen H-Y (2004). Antagonistic activity against Helicobacter pylori infection in vitro by a strain of Enterococcus faecium TM39. Int J Food Microbiol.

[82] Hurduc V, Plesca D, Dragomir D, Sajin M, Vandenplas Y (2009). A randomized, open trial evaluating the effect of Saccharomyces boulardii on the eradication rate of Helicobacter pylori infection in children. Acta Paediatr.

[83] Scaccianoce G, Zullo A, Hassan C, Gentili F, Cristofari F, Cardinale V (2008). Triple therapies plus different probiotics for. Eur Rev Med Pharmacol Sci.

[84] Do AD, Chang CC, Su CH, Hsu YM (2021). Lactobacillus rhamnosus JB3 inhibits Helicobacter pylori infection through multiple molecular actions. Helicobacter.

[85] Karbalaei M, Keikha M (2020). Potential association between the hopQ alleles of Helicobacter pylori and gastrointestinal diseases: A systematic review and meta-analysis. Meta Gene..

[86] Mukai T, Asasaka T, Sato E, Mori K, Matsumoto M, Ohori H (2002). Inhibition of binding of Helicobacter pylori to the glycolipid receptors by probiotic Lactobacillus reuteri. FEMS Immunol Med Microbiol.

[87] Sakarya S, Gunay N (2014). S accharomyces boulardii expresses neuraminidase activity selective for α2, 3-linked sialic acid that decreases H elicobacter pylori adhesion to host cells. APMIS.

[88] Canducci F, Armuzzi A, Cremonini F, Cammarota G, Bartolozzi F, Pola P (2000). A lyophilized and inactivated culture of Lactobacillus acidophilus increases Helicobacter pylori eradication rates. Aliment Pharmacol Ther.

[89] Hsieh PS, Tsai YC, Chen YC, Teh SF, Ou CM, King VAE (2012). Eradication of Helicobacter pylori Infection by the Probiotic Strains Lactobacillus johnsonii MH-68 and L. salivarius ssp. salicinius AP-32. Helicobacter.

[90] Lee Y-H (2004). Weissella confusa strain PL9001 inhibits growth and adherence of genitourinary pathogens. J Microbiol Biotechnol.

[91] Myllyluoma E, Kajander K, Mikkola H, Kyrönpalo S, Rasmussen M, Kankuri E (2007). Probiotic intervention decreases serum gastrin-17 in Helicobacter pylori infection. Dig Liver Dis.

[92] Khan S, Moore RJ, Stanley D, Chousalkar KK (2020). The gut microbiota of laying hens and its manipulation with prebiotics and probiotics to enhance gut health and food safety. Appl Environ Microbiol..

[93] Van den Brink G, Tytgat K, Van der Hulst R, Van der Loos C, Einerhand A, Büller H (2000). H pylori colocalises with MUC5AC in the human stomach. Gut.

[94] Mack DR, Michail S, Wei S, McDougall L, Hollingsworth MA. Probiotics inhibit enteropathogenic E. coli adherence in vitro by inducing intestinal mucin gene expression. Am J Physiol Gastrointest Liver Physiol. 1999;276(4):G941–50. 10.1152/ajpgi.1999.276.4.G941 10198338

[95] Pantoflickova D, Corthesy-Theulaz I, Dorta G, Stolte M, Isler P, Rochat F (2003). Favourable effect of regular intake of fermented milk containing Lactobacillus johnsonii on Helicobacter pylori associated gastritis. Aliment Pharmacol Ther.

[96] Schiffrin E, Blum S (2002). Interactions between the microbiota and the intestinal mucosa. Eur J Clin Nutr.

[97] Angelakis E, Merhej V, Raoult D (2013). Related actions of probiotics and antibiotics on gut microbiota and weight modification. Lancet Infect Dis.

[98] Khoshbin Z, Verdian A, Housaindokht MR, Izadyar M, Rouhbakhsh Z (2018). Aptasensors as the future of antibiotics test kits-a case study of the aptamer application in the chloramphenicol detection. Biosens Bioelectron.

[99] Procópio REDL, Silva IRD, Martins MK, Azevedo JLD, Araújo JMD (2012). Antibiotics produced by Streptomyces. Braz J Infect Dis.

[100] Rietkötter E, Hoyer D, Mascher T (2008). Bacitracin sensing in Bacillus subtilis. Mol Microbiol.

[101] Algood HMS, Cover TL (2006). Helicobacter pylori persistence: an overview of interactions between H. pylori and host immune defenses. Clin Microbiol Rev.

[102] Boyanova L, Gergova G, Markovska R, Yordanov D, Mitov I (2017). Bacteriocin-like inhibitory activities of seven Lactobacillus delbrueckii subsp. bulgaricus strains against antibiotic susceptible and resistant Helicobacter pylori strains. Lett Appl Microbiol.

[103] Negash AW, Tsehai BA (2020). Current applications of Bacteriocin. Int J Microbiol..

[104] Rezaee P, Kermanshahi RK, Falsafi T (2019). Antibacterial activity of lactobacilli probiotics on clinical strains of Helicobacter pylori. Iran J Basic Med Sci.

[105] Collado M, Gonzalez A, Gonzalez R, Hernandez M, Ferrus M, Sanz Y (2005). Antimicrobial peptides are among the antagonistic metabolites produced by Bifidobacterium against Helicobacter pylori. Int J Antimicrob Agents.

[106] Enany S, Abdalla S (2015). In vitro antagonistic activity of Lactobacillus casei against Helicobacter pylori. Braz J Microbiol.

[107] Fujita Y, Yamaguchi K, Kamegaya T, Sato H, Semura K, Mutoh K (2005). A novel mechanism of autolysis in Helicobacter pylori: possible involvement of peptidergic substances. Helicobacter.

[108] Urrutia-Baca VH, Escamilla-García E, de la Garza-Ramos MA, Tamez-Guerra P, Gomez-Flores R, Urbina-Ríos CS (2018). In vitro antimicrobial activity and downregulation of virulence gene expression on Helicobacter pylori by reuterin. Probiotics Antimicrob Proteins.

[109] Holz C, Busjahn A, Mehling H, Arya S, Boettner M, Habibi H (2015). Significant reduction in Helicobacter pylori load in humans with non-viable Lactobacillus reuteri DSM17648: a pilot study. Probiot Antimicrob Proteins.

[110] Chen X, Tian F, Liu X, Zhao J, Zhang H-P, Zhang H (2010). In vitro screening of lactobacilli with antagonistic activity against Helicobacter pylori from traditionally fermented foods. J Dairy Sci.

[111] Blum S, Haller D, Pfeifer A, Schiffrin EJ (2002). Probiotics and immune response. Clin Rev Allergy Immunol.

[112] Yang Y-J, Chuang C-C, Yang H-B, Lu C-C, Sheu B-S (2012). Lactobacillus acidophilus ameliorates H. pylori-induced gastric inflammation by inactivating the Smad7 and NFκB pathways. BMC Microbiol.

[113] Lam EK, Tai EK, Koo MW, Wong HP, Wu WK, Yu L (2007). Enhancement of gastric mucosal integrity by Lactobacillus rhamnosus GG. Life Sci.

[114] Jackson L, Wu K, Mahida Y, Jenkins D, Hawkey C (2000). Cyclooxygenase (COX) 1 and 2 in normal, inflamed, and ulcerated human gastric mucosa. Gut.

[115] Keikha M, Eslami M, Yousefi B, Ghasemian A, Karbalaei M (2019). Potential antigen candidates for subunit vaccine development against Helicobacter pylori infection. J Cell Physiol.

[116] Sutton P, Boag JM (2019). Status of vaccine research and development for Helicobacter pylori. Vaccine.

[117] Qureshi N, Li P, Gu Q (2019). Probiotic therapy in Helicobacter pylori infection: a potential strategy against a serious pathogen?. Appl Microbiol Biotechnol.

[118] Song D, Gu Q (2009). Surface expression of Helicobacter pylori urease subunit B gene E fragment on Lactococcus lactis by means of the cell wall anchor of Staphylococcus aureus protein A. Biotech Lett.

[119] Zhou Z, Gong S, Li X-M, Yang Y, Guan R, Zhou S (2015). Expression of Helicobacter pylori urease B on the surface of Bacillus subtilis spores. J Med Microbiol.

[120] Li X, Xing Y, Guo L, Lv X, Song H, Xi T (2014). Oral immunization with recombinant Lactococcus lactis delivering a multi-epitope antigen CTB-UE attenuates Helicobacter pylori infection in mice. Pathog Dis.

[121] Peng X, Zhang R, Duan G, Wang C, Sun N, Zhang L (2018). Production and delivery of Helicobacter pylori NapA in Lactococcus lactis and its protective efficacy and immune modulatory activity. Sci Rep.

[122] Ushiyama A, Tanaka K, Aiba Y, Shiba T, Takagi A, Mine T (2003). Lactobacillus gasseri OLL2716 as a probiotic in clarithromycin-resistant Helicobacter pylori infection. J Gastroenterol Hepatol.

[123] Peña JA, Rogers AB, Ge Z, Ng V, Li SY, Fox JG (2005). Probiotic Lactobacillus spp. diminish Helicobacter hepaticus-induced inflammatory bowel disease in interleukin-10-deficient mice. Infect Immunity.

[124] Sgouras DN, Panayotopoulou EG, Martinez-Gonzalez B, Petraki K, Michopoulos S, Mentis Α (2005). Lactobacillus johnsonii La1 attenuates Helicobacter pylori-associated gastritis and reduces levels of proinflammatory chemokines in C57BL/6 mice. Clin Vaccine Immunol.

[125] Moyat M, Velin D (2014). Immune responses to Helicobacter pylori infection. World J Gastroenterol WJG.

[126] Salama NR, Hartung ML, Müller A (2013). Life in the human stomach: persistence strategies of the bacterial pathogen Helicobacter pylori. Nat Rev Microbiol.

[127] Wang X, Wang B, Gao W, An Y, Dong G, Jia J (2021). Helicobacter pylori inhibits autophagic flux and promotes its intracellular survival and colonization by down-regulating SIRT1. J Cell Mol Med.

[128] Chang C-C, Kuo W-S, Chen Y-C, Perng C-L, Lin H-J, Ou Y-H (2016). Fragmentation of CagA reduces hummingbird phenotype induction by helicobactor pylori. PLoS ONE.

[129] Keikha M, Karbalaei M (2021). EPIYA motifs of Helicobacter pylori cagA genotypes and gastrointestinal diseases in the Iranian population: a systematic review and meta-analysis. New Microbes New Infect..

[130] Wang H-P, Zhu Y-L, Shao W (2013). Role of Helicobacter pylori virulence factor cytotoxin-associated gene A in gastric mucosa-associated lymphoid tissue lymphoma. World J Gastroenterol WJG.

[131] Sugimoto M, Murata M, Yamaoka Y (2020). Chemoprevention of gastric cancer development after Helicobacter pylori eradication therapy in an East Asian population: meta-analysis. World J Gastroenterol.

[132] Farinha P, Gascoyne RD (2005). Helicobacter pylori and MALT lymphoma. Gastroenterology.

[133] Du M-Q, editor MALT lymphoma: A paradigm of NF-κB dysregulation. Seminars in cancer biology; 2016: Elsevier. 10.1016/j.semcancer.2016.07.003 27452667

[134] Ailioaie LM, Litscher G (2021). Probiotics, photobiomodulation, and disease management: controversies and challenges. Int J Mol Sci.

[135] Fujimura S, Watanabe A, Kimura K, Kaji M (2012). Probiotic mechanism of Lactobacillus gasseri OLL2716 strain against Helicobacter pylori. J Clin Microbiol.

[136] Espinoza JL, Matsumoto A, Tanaka H, Matsumura I (2018). Gastric microbiota: an emerging player in Helicobacter pylori-induced gastric malignancies. Cancer Lett.

[137] Sgouras DN, Panayotopoulou EG, Martinez-Gonzalez B, Petraki K, Michopoulos S, Mentis Α (2005). Lactobacillus johnsonii La1 attenuates Helicobacter pylori-associated gastritis and reduces levels of proinflammatory chemokines in C57BL/6 mice. Clin Diagn Lab Immunol.

[138] Brzozowski T, Konturek PC, Mierzwa M, Drozdowicz D, Bielanski W, Kwiecien S (2006). Effect of probiotics and triple eradication therapy on the cyclooxygenase (COX)-2 expression, apoptosis, and functional gastric mucosal impairment in Helicobacter pylori-infected Mongolian gerbils. Helicobacter.

[139] Chenoll E, Casinos B, Bataller E, Astals P, Echevarría J, Iglesias JR (2011). Novel probiotic Bifidobacterium bifidum CECT 7366 strain active against the pathogenic bacterium Helicobacter pylori. Appl Environ Microbiol.

[140] Kuo C-H, Wang SS, Lu C-Y, Hu H-M, Kuo F-C, Weng B-C (2013). Long-term use of probiotic-containing yogurts is a safe way to prevent Helicobacter pylori: based on a Mongolian gerbil's model. Biochem Res Int..

[141] Kaur B, Garg N, Sachdev A, Kumar B (2014). Effect of the oral intake of probiotic Pediococcus acidilactici BA28 on Helicobacter pylori causing peptic ulcer in C57BL/6 mice models. Appl Biochem Biotechnol.

[142] Kim J-E, Kim M-S, Yoon Y-S, Chung M-J, Yum D-Y (2014). Use of selected lactic acid bacteria in the eradication of Helicobacter pylori infection. J Microbiol.

[143] Zaman C, Osaki T, Hanawa T, Yonezawa H, Kurata S, Kamiya S (2014). Analysis of the microbial ecology between Helicobacter pylori and the gastric microbiota of Mongolian gerbils. J Med Microbiol.

[144] Matsui H, Takahashi T, Øverby A, Murayama SY, Yoshida H, Yamamoto Y (2015). Mouse models for assessing the protective efficacy of Lactobacillus gasseri SBT 2055 against helicobacter suis infection associated with the development of gastric mucosa-associated lymphoid tissue lymphoma. Helicobacter.

[145] Yu H-J, Liu W, Chang Z, Shen H, He L-J, Wang S-S (2015). Probiotic BIFICO cocktail ameliorates Helicobacter pylori induced gastritis. World J Gastroenterol WJG.

[146] Pan M, Wan C, Xie Q, Huang R, Tao X, Shah NP (2016). Changes in gastric microbiota induced by Helicobacter pylori infection and preventive effects of Lactobacillus plantarum ZDY 2013 against such infection. J Dairy Sci.

[147] Afsahi A, Mahmoudi H, Ebrahimi A, Aeini Z, Esmaeili D (2018). Evaluation of the effect of lactobacillus planetarium probiotics produced from broad bean seed in prevention of helicobacter pylori in stomach tissue of C57BL/6 mice. J Cancer Sci Ther.

[148] Chen ME, Su CH, Yang JS, Lu CC, Hou YC, Wu JB (2018). Baicalin, baicalein, and lactobacillus rhamnosus JB3 alleviated Helicobacter pylori infections in vitro and in vivo. J Food Sci.

[149] Merino J, García A, Pastene E, Salas A, Saez K, González C (2018). Lactobacillus fermentum UCO-979C strongly inhibited Helicobacter pylori SS1 in Meriones unguiculatus. Beneficial Microbes.

[150] Lin C-C, Huang W-C, Su C-H, Lin W-D, Wu W-T, Yu B (2020). Effects of multi-strain probiotics on immune responses and metabolic balance in helicobacter pylori-infected mice. Nutrients.

[151] Cruchet S, Obregon MC, Salazar G, Diaz E, Gotteland M (2003). Effect of the ingestion of a dietary product containing Lactobacillus johnsonii La1 on Helicobacter pylori colonization in children. Nutrition.

[152] Gotteland M, Poliak L, Cruchet S, Brunser O (2005). Effect of regular ingestion of Saccharomyces boulardii plus inulin or Lactobacillus acidophilus LB in children colonized by Helicobacter pylori. Acta Paediatr.

[153] Patel A, Shah N, Prajapati J (2014). Clinical application of probiotics in the treatment of Helicobacter pylori infection—a brief review. J Microbiol Immunol Infect.

[154] Bagarolli RA, Tobar N, Oliveira AG, Araújo TG, Carvalho BM, Rocha GZ (2017). Probiotics modulate gut microbiota and improve insulin sensitivity in DIO mice. J Nutr Biochem.

[155] Marighela TF, Arismendi MI, Marvulle V, Brunialti MKC, Salomão R, Kayser C (2019). Effect of probiotics on gastrointestinal symptoms and immune parameters in systemic sclerosis: a randomized placebo-controlled trial. Rheumatology.

[156] Sýkora J, Valecková K, Amlerová J, Siala K, Dedek P, Watkins S (2005). Effects of a specially designed fermented milk product containing probiotic Lactobacillus casei DN-114 001 and the eradication of H. pylori in children: a prospective randomized double-blind study. J Clin Gastroenterol.

[157] Goldman CG, Barrado DA, Balcarce N, Rua EC, Oshiro M, Calcagno ML (2006). Effect of a probiotic food as an adjuvant to triple therapy for eradication of Helicobacter pylori infection in children. Nutrition.

[158] Lionetti E, Miniello V, Castellaneta S, Magista A, De Canio A, Maurogiovanni G (2006). Lactobacillus reuteri therapy to reduce side-effects during anti-Helicobacter pylori treatment in children: a randomized placebo controlled trial. Aliment Pharmacol Ther.

[159] Gotteland M, Andrews M, Toledo M, Muñoz L, Caceres P, Anziani A (2008). Modulation of Helicobacter pylori colonization with cranberry juice and Lactobacillus johnsonii La1 in children. Nutrition.

[160] Szajewska H, Albrecht P, Topczewska-Cabanek A (2009). Randomized, double-blind, placebo-controlled trial: effect of lactobacillus GG supplementation on Helicobacter pylori eradication rates and side effects during treatment in children. J Pediatr Gastroenterol Nutr.

[161] Boonyaritichaikij S, Kuwabara K, Nagano J, Kobayashi K, Koga Y (2009). Long-term administration of probiotics to asymptomatic pre-school children for either the eradication or the prevention of Helicobacter pylori infection. Helicobacter.

[162] Tolone S, Pellino V, Vitaliti G, Tolone C (2012). Evaluation of Helicobacter Pylori eradication in pediatric patients by triple therapy plus lactoferrin and probiotics compared to triple therapy alone. Ital J Pediatr.

[163] Zhao H-M, Ou-Yang H-J, Duan B-P, Xu B, Chen Z-Y, Tang J, et al. Clinical effect of triple therapy combined with Saccharomyces boulardii in the treatment of Helicobacter pylori infection in children. Zhongguo dang dai er ke za zhi= Chin J Contemp Pediatr. 2014;16(3):230–3. 24661511

[164] Wang Y-h, Huang Y. Effect of Lactobacillus acidophilus and Bifidobacterium bifidum supplementation to standard triple therapy on Helicobacter pylori eradication and dynamic changes in intestinal flora. World J Microbiol Biotechnol. 2014;30(3):847–53. 10.1007/s11274-013-1490-2 24233772

[165] Akcam M, Koca T, Salman H, Karahan N (2015). The effects of probiotics on treatment of Helicobacter pylori eradication in children. Saudi Med J.

[166] Zhu X-L, Liu Z, Wu Z-Q, Li D, Jiang A-P, Yu G-X. Clinical effects of different therapeutic regimens for Helicobacter pylori infection in children. Zhongguo Dang dai er ke za zhi= Chin J Contemp Pediatr. 2017;19(6):672–6. 10.7499/j.issn.1008-8830.2017.06.012 PMC7390303 28606235

[167] Li S, Huang X-l, Sui J-z, Chen S-y, Xie Y-t, Deng Y, et al. Meta-analysis of randomized controlled trials on the efficacy of probiotics in Helicobacter pylori eradication therapy in children. Eur J Pediatr. 2014;173(2):153–61. 10.1007/s00431-013-2220-3 24323343

[168] Fang H-R, Zhang G-Q, Cheng J-Y, Li Z-Y (2019). Efficacy of Lactobacillus-supplemented triple therapy for Helicobacter pylori infection in children: a meta-analysis of randomized controlled trials. Eur J Pediatr.

[169] Tong J, Ran Z, Shen J, Zhang C, Xiao S (2007). Meta-analysis: the effect of supplementation with probiotics on eradication rates and adverse events during Helicobacter pylori eradication therapy. Aliment Pharmacol Ther.

[170] Sachdeva A, Nagpal J (2009). Effect of fermented milk-based probiotic preparations on Helicobacter pylori eradication: a systematic review and meta-analysis of randomized-controlled trials. Eur J Gastroenterol Hepatol.

[171] Zou J, Dong J, Yu X (2009). Meta-analysis: Lactobacillus containing quadruple therapy versus standard triple first-line therapy for Helicobacter pylori eradication. Helicobacter.

[172] Szajewska H, Horvath A, Piwowarczyk A (2010). Meta-analysis: the effects of Saccharomyces boulardii supplementation on Helicobacter pylori eradication rates and side effects during treatment. Aliment Pharmacol Ther.

[173] Zheng X, Lyu L, Mei Z (2013). Lactobacillus-containing probiotic supplementation increases Helicobacter pylori eradication rate: evidence from a meta-analysis. Rev Esp Enferm Dig.

[174] Wang Z-H, Gao Q-Y, Fang J-Y (2013). Meta-analysis of the efficacy and safety of Lactobacillus-containing and Bifidobacterium-containing probiotic compound preparation in Helicobacter pylori eradication therapy. J Clin Gastroenterol.

[175] Zhu R, Chen K, Zheng Y-Y, Zhang H-W, Wang J-S, Xia Y-J (2014). Meta-analysis of the efficacy of probiotics in Helicobacter pylori eradication therapy. World J Gastroenterol WJG.

[176] Dang Y, Reinhardt JD, Zhou X, Zhang G (2014). The effect of probiotics supplementation on Helicobacter pylori eradication rates and side effects during eradication therapy: a meta-analysis. PLoS ONE.

[177] Zhang M-M, Qian W, Qin Y-Y, He J, Zhou Y-H (2015). Probiotics in Helicobacter pylori eradication therapy: a systematic review and meta-analysis. World J Gastroenterol WJG.

[178] Lu C, Sang J, He H, Wan X, Lin Y, Li L (2016). Probiotic supplementation does not improve eradication rate of Helicobacter pylori infection compared to placebo based on standard therapy: a meta-analysis. Sci Rep.

[179] Lü M, Yu S, Deng J, Yan Q, Yang C, Xia G (2016). Efficacy of probiotic supplementation therapy for Helicobacter pylori eradication: a meta-analysis of randomized controlled trials. PLoS ONE.

[180] Si X, Lan Y, Qiao L (2017). A meta-analysis of randomized controlled trials of bismuth-containing quadruple therapy combined with probiotic supplement for eradication of Helicobacter pylori. Zhonghua Nei Ke Za Zhi.

[181] Losurdo G, Cubisino R, Barone M, Principi M, Leandro G, Ierardi E (2018). Probiotic monotherapy and Helicobacter pylori eradication: a systematic review with pooled-data analysis. World J Gastroenterol.

[182] Shi X, Zhang J, Mo L, Shi J, Qin M, Huang X (2019). Efficacy and safety of probiotics in eradicating Helicobacter pylori: a network meta-analysis. Medicine..

[183] Yu M, Zhang R, Ni P, Chen S, Duan G (2019). Efficacy of Lactobacillus-supplemented triple therapy for H. pylori eradication: a meta-analysis of randomized controlled trials. PLoS ONE.

[184] Pourmasoumi M, Najafgholizadeh A, Hadi A, Mansour-Ghanaei F, Joukar F (2019). The effect of synbiotics in improving Helicobacter pylori eradication: a systematic review and meta-analysis. Complement Ther Med.

[185] Zhou BG, Chen LX, Li B, Wan LY, Ai YW (2019). Saccharomyces boulardii as an adjuvant therapy for Helicobacter pylori eradication: a systematic review and meta-analysis with trial sequential analysis. Helicobacter.

[186] Wang F, Feng J, Chen P, Liu X, Ma M, Zhou R (2017). Probiotics in Helicobacter pylori eradication therapy: systematic review and network meta-analysis. Clin Res Hepatol Gastroenterol.

[187] Abadi ATB (2016). Vaccine against Helicobacter pylori: Inevitable approach. World J Gastroenterol.

[188] Savage DC (1977). Microbial ecology of the gastrointestinal tract. Annu Rev Microbiol.

[189] Ley RE, Hamady M, Lozupone C, Turnbaugh PJ, Ramey RR, Bircher JS, et al. Evolution of mammals and their gut microbes. Science. 2008;320(5883):1647–51. 10.1126/science.1155725 PMC2649005 18497261

[190] Weingarden AR, Vaughn BP (2017). Intestinal microbiota, fecal microbiota transplantation, and inflammatory bowel disease. Gut Microbes.

[191] Zeng Y, Luo M, Pan L, Chen Y, Guo S, Luo D, et al. Vitamin D signaling maintains intestinal innate immunity and gut microbiota: potential intervention for metabolic syndrome and NAFLD. American Journal of Physiology-Gastrointestinal and Liver Physiology. 2020;318(3):G542-G53. 10.1152/ajpgi.00286.2019 PMC7099486 31984787

[192] Hold GL (2016). Gastrointestinal microbiota and colon cancer. Dig Dis.

[193] Myllyluoma E, Ahlroos T, Veijola L, Rautelin H, Tynkkynen S, Korpela R (2007). Effects of anti-Helicobacter pylori treatment and probiotic supplementation on intestinal microbiota. Int J Antimicrob Agents.

[194] Ye Q, Shao X, Shen R, Chen D, Shen J. Changes in the human gut microbiota composition caused by Helicobacter pylori eradication therapy: A systematic review and meta‐analysis. Helicobacter. 2020;25(4):e12713. 10.1111/hel.12713 32515529

[195] Oh B, Kim JW, Kim BS (2016). Changes in the functional potential of the gut microbiome following probiotic supplementation during Helicobacter pylori treatment. Helicobacter.

[196] Wang Z-J, Chen X-F, Zhang Z-X, Li Y-C, Deng J, Tu J (2017). Effects of anti-Helicobacter pylori concomitant therapy and probiotic supplementation on the throat and gut microbiota in humans. Microb Pathog.

[197] Cárdenas PA, Garcés D, Prado-Vivar B, Flores N, Fornasini M, Cohen H (2020). Effect of Saccharomyces boulardii CNCM I-745 as complementary treatment of Helicobacter pylori infection on gut microbiome. Eur J Clin Microbiol Infect Dis.

[198] Gao C, Major A, Rendon D, Lugo M, Jackson V, Shi Z (2015). Histamine H2 receptor-mediated suppression of intestinal inflammation by probiotic Lactobacillus reuteri. MBio..

